# Genomic selection for spot blotch in bread wheat breeding panels, full-sibs and half-sibs and index-based selection for spot blotch, heading and plant height

**DOI:** 10.1007/s00122-022-04087-y

**Published:** 2022-04-13

**Authors:** Philomin Juliana, Xinyao He, Jesse Poland, Krishna K. Roy, Paritosh K. Malaker, Vinod K. Mishra, Ramesh Chand, Sandesh Shrestha, Uttam Kumar, Chandan Roy, Navin C. Gahtyari, Arun K. Joshi, Ravi P. Singh, Pawan K. Singh

**Affiliations:** 1 Borlaug Institute for South Asia (BISA), Ludhiana, Punjab India; 2 International Maize and Wheat Improvement Center (CIMMYT), Apdo. Postal 6-641, 06600 Mexico, DF Mexico; 3 Biological and Environmental Science and Engineering Division, King Abdullah University of Science and Technology (KAUST), Thuwal, Saudi Arabia; 4 Bangladesh Wheat and Maize Research Institute, Nashipur, Dinajpur, 5200 Bangladesh; 5 Institute of Agricultural Sciences, Banaras Hindu University, Varanasi, Uttar Pradesh India; 6 Department of Plant Pathology, Wheat Genetics Resource Center, Kansas State University, Manhattan, KS USA; 7 Department of Plant Breeding and Genetics, Bihar Agricultural University, Sabour, Bihar 813210 India; 8 ICAR–Vivekanand Parvatiya Krishi Anushandhan Sansthan, Almora, Uttarakhand 263601 India; 9 CIMMYT-India, NASC Complex, DPS Marg, New Delhi, India

## Abstract

**Key message:**

Genomic selection is a promising tool to select for spot blotch resistance and index-based selection can simultaneously select for spot blotch resistance, heading and plant height.

**Abstract:**

A major biotic stress challenging bread wheat production in regions characterized by humid and warm weather is spot blotch caused by the fungus *Bipolaris sorokiniana*. Since genomic selection (GS) is a promising selection tool, we evaluated its potential for spot blotch in seven breeding panels comprising 6736 advanced lines from the International Maize and Wheat Improvement Center. Our results indicated moderately high mean genomic prediction accuracies of 0.53 and 0.40 within and across breeding panels, respectively which were on average 177.6% and 60.4% higher than the mean accuracies from fixed effects models using selected spot blotch loci. Genomic prediction was also evaluated in full-sibs and half-sibs panels and sibs were predicted with the highest mean accuracy (0.63) from a composite training population with random full-sibs and half-sibs. The mean accuracies when full-sibs were predicted from other full-sibs within families and when full-sibs panels were predicted from other half-sibs panels were 0.47 and 0.44, respectively. Comparison of GS with phenotypic selection (PS) of the top 10% of resistant lines suggested that GS could be an ideal tool to discard susceptible lines, as greater than 90% of the susceptible lines discarded by PS were also discarded by GS. We have also reported the evaluation of selection indices to simultaneously select non-late and non-tall genotypes with low spot blotch phenotypic values and genomic-estimated breeding values. Overall, this study demonstrates the potential of integrating GS and index-based selection for improving spot blotch resistance in bread wheat.

**Supplementary Information:**

The online version contains supplementary material available at 10.1007/s00122-022-04087-y.

## Introduction

One of the major biotic stresses challenging bread wheat (*Triticum aestivum*) production in regions characterized by humid and warm weather conditions is spot blotch (also known as Helminthosporium leaf blight), caused by the fungus *Bipolaris sorokiniana* (Sacc.) Shoemaker (teleomorph: *Cochliobolus sativus* (Ito and Kuribayashi) Drechsler ex Dastur). The disease is widely prevalent in the eastern Gangetic plains of India, the Terai region of Nepal, Bangladesh, parts of Southeast Asia, Africa and Latin America (van Ginkel and Rajaram 1998; Joshi et al. 2007; Gupta et al. 2018) and remains as a persistent threat to the livelihoods of numerous farmers in these regions (Dubin and van Ginkel 1991; Saari et al. 1998; Duveiller and Sharma 2009; Singh et al. 2018a). Several cultural, agronomic and chemical control approaches have been proposed for the management of spot blotch in wheat, but none of them are completely effective (Duveiller et al. 2005; Pandey et al. 2005; Sharma and Duveiller 2006a; Sharma et al. 2006; Duveiller and Sharma 2009). Hence, an integrated approach combining the best cultural and agronomic management practices with acceptable fungicide control along with the deployment of varieties with genetic resistance to spot blotch has been recommended as the most ideal disease management strategy (Joshi et al. 2004a; Sharma and Duveiller 2006b; Duveiller and Sharma 2009).

Genetic resistance to spot blotch was initially suggested to be qualitative, involving one or few dominant (Srivastava et al. 1971), partial dominant (Adlakha 1984; Velázquez Cruz et al. 1994; Sharma and Bhatta 1999; Neupane et al. 2007) or recessive genes (Singh et al. 1998a, 2000; Bhushan et al. 2002; Ragiba et al. 2004). Four spot blotch resistance genes including *Sb*1*, Sb*2*, Sb*3 and *Sb*4 have been identified so far (Lillemo et al. 2013; Kumar et al. 2015; Lu et al. 2016; Zhang et al. 2020). Meanwhile, other studies have demonstrated the quantitative genetic control of resistance to spot blotch involving many loci with small effects (Sharma et al. 1997; Joshi et al. 2004b; Juliana et al. 2022). However, breeding for quantitative spot blotch resistance using conventional selection methods has been slow due to the limited efficiency to select at multiple minor resistance loci (Joshi et al. 2004b; Sharma et al. 2007; Gupta et al. 2018).

A promising selection tool that utilizes the best estimates of the effects of dense genome-wide molecular markers on the trait vs specific QTL and enables selection on multiple loci is genomic selection (GS) (Meuwissen et al. 2001, 2013; Heffner et al. 2009; Lorenz et al. 2011). The advantage of GS over marker-assisted selection based on few markers is that GS has the potential to increase selection accuracy by fitting all the marker effects simultaneously including those tagging small-effect QTL (Jannink et al. 2010; Meuwissen et al. 2013). Using GS, individuals that have only been genotyped but not phenotyped (referred to as selection candidates or validation population) can be selected based on the genomic-estimated breeding values (GEBVs) obtained from prediction models that are trained using lines that have both phenotyping and genotyping data (referred to as the training population) (Lorenz et al. 2011). The ability to use GS for selecting lines that have not been phenotyped can reduce the breeding cycle time and cost, increase the selection intensity and subsequently increase the rate of genetic gain for traits (Heffner et al. 2010; Poland and Rutkoski 2016; Voss-Fels et al. 2019).

Given the potential of GS to improve quantitative traits, it has been extensively evaluated for various diseases in wheat (Rutkoski et al. 2012; Juliana et al. 2017a, b, 2019; Herter et al. 2019; Emebiri et al. 2021). However, a comprehensive evaluation of GS for spot blotch in bread wheat is not available, and hence the key objective of this study was to evaluate the potential of GS for spot blotch breeding using a large population comprising 6736 advanced breeding lines from the International Maize and Wheat Improvement Center (CIMMYT). The GS scenarios that we evaluated involved predictions within breeding panels/years using fivefold cross validations (Lorenz et al. 2011) and predictions across breeding panels using all other panels as training sets. For both these scenarios, we tested the hypothesis that genome-wide marker relationship*-*based predictions using the genomic-best linear unbiased prediction (GBLUP) model (de los Campos et al. 2013; Habier et al. 2013) performs better than predictions using only few significant markers as fixed effects. We also aimed at understanding the advantage of using a combined GBLUP and fixed effects model (GBLUP + fixed effects) over the GBLUP model and comparing GS for spot blotch with phenotypic selection (PS) in both the GS scenarios.

While the advanced wheat breeding populations from CIMMYT comprise families with only few full-sibs and half-sibs (Juliana et al. 2020), designing a GS-based breeding strategy for spot blotch resistance in seed-limiting early-generations of the breeding cycle will require a good understanding of the genomic predictability in large full-sibs and half-sibs populations. Hence, we used four full-sibs populations with one common parent for evaluating genomic prediction with different training populations. In these populations, we also compared genomic prediction accuracies with accuracies from fixed effects models and the combined GBLUP + fixed effects models, and selections made using PS and GS.

A key concern in integrating GS with PS in selection decisions for spot blotch resistance is the lack of a simple and efficient framework that can facilitate breeders to simultaneously select on both. In addition, while some studies have indicated that selection for spot blotch resistance can be independent of days to heading and plant height (Joshi et al. 2002; Singh et al. 2015), their negative correlation in other studies has complicated the selection for spot blotch resistance, which also involves selecting for early-maturing and short plants (Dubin and Rajaram 1996; Sharma et al. 1997; Dubin et al. 1998; Saari et al. 1998). In this regard, selection index is an important tool that can combine information on multiple traits into a single value, maximize the selection response and facilitate breeders in the selection of individuals based on their ranking or predicted net genetic merit (Smith 1936; Hazel and Lush 1942; Hazel 1943; Céron-Rojas and Crossa 2018). The use of a selection index that could facilitate eliminating lines with late heading and low spot blotch severity (possibly due to disease escape) was suggested by Sharma et al. (1997). A simple selection index combining the spot blotch disease severity, days to heading/maturity and thousand kernel weight was reported by Sharma and Duveiller (2003) and Sharma and Duveiller (2006a; b). However, a selection index combining spot blotch phenotypic values with the GEBVs, days to heading and plant height has not been reported till date, and hence another key objective of this study was to evaluate index-based selection to select simultaneously for these traits.

## Materials and methods

### Populations and spot blotch evaluation environments

The breeding population used in this study comprised seven panels, each with 1092 advanced breeding lines from the CIMMYT bread wheat program’s stage 2 yield trial nurseries. These breeding lines were developed using the selected bulk breeding approach (Singh et al. 1998b). In this approach, plants selected visually for agronomic traits and disease resistance are bulked in the early-generations of the breeding cycle, until the head-rows stage where individual plants are derived from the F_4_, F_5_ or F_6_ generations. From the head-rows, about 9000 lines are selected for the stage 1 of yield testing. Phenotypic data for the stage 1 yield trial lines is used to further select lines with high grain yield, acceptable agronomic type, phenology and end-use quality, and good resistance to rusts to constitute the stage 2 yield trial nurseries. The stage 2 yield trial lines from each cycle were evaluated for spot blotch in CIMMYT’s spot blotch screening station at Agua Fria, Mexico (19° 59′ N, 97° 50′ W) in subsequent crop cycles from 2013–2014 to 2019–2020 and are referred to by the harvest year and site of evaluation (for example: panel 2014 Agua Fria).

The full-sibs populations used in this study included Bartai × Ciano T79 (BC), Cascabel × Ciano T79 (CC), Kath × Ciano T79 (KC) and Wuya × Ciano T79 (WC) (Singh et al. 2018a; He et al. 2020; Gahtyari et al. 2021; Roy et al. 2021). Each of them comprised 231 progenies, obtained from crossing a susceptible cultivar Ciano T79 (BUCKY/(SIB)MAYA-74/4/BLUEBIRD//HD-832.5.5/OLESEN/3/CIANO-67/PENJAMO-62) with four resistant cultivars including: (1) Bartai (BABAX/LR42//BABAX/3/ERA F2000) (2) Casacabel (SOKOLL//W15.92/WEEBILL1) (3) Kath (WHEATEAR/KRONSTAD F2004) and (4) Wuya (WAXWING*2/CIRCUS). These populations were developed using the single seed descent method, where a single seed from individual F_2_ progenies in each population was advanced to generate F_2:7_ recombinant inbred lines.

All the full-sibs populations were evaluated for spot blotch in Agua Fria during the 2012–2013 to 2014–2015 cycles and are referred to by the name of the population, the harvest year and the site of evaluation as follows: (1) BC 2013 Agua Fria (2) BC 2014 Agua Fria (3) BC 2015 Agua Fria (4) CC 2013 Agua Fria (5) CC 2014 Agua Fria (6) CC 2015 Agua Fria (7) KC 2013 Agua Fria (8) KC 2014 Agua Fria (9) KC 2015 Agua Fria (10) WC 2013 Agua Fria (11) WC 2014 Agua Fria (12) WC 2015 Agua Fria. In addition, some full-sibs populations were also evaluated in two South Asian spot blotch hot spots including Varanasi, India (25° 16′ N and 82° 59′ E) and Dinajpur, Bangladesh (25° 38′ N and 88° 38′ E). The CC population was evaluated for spot blotch in Varanasi in the 2012–2013 cycle (CC 2013 Varanasi), the KC population was evaluated in Dinajpur in the 2012–2013 cycle (KC 2013 Dinajpur) and the WC population was evaluated in Dinajpur in the 2012–2013 and 2013–2014 cycles (WC 2013 Dinajpur and WC 2014 Dinajpur).

### Phenotyping data

Field response to spot blotch in Agua Fria in all the seven breeding panels and the four full-sibs panels was evaluated on lines that were planted in November and harvested in March each season. The lines were sown in plots using a randomized complete block design. Each plot comprised two rows that were 1 m in length, where the lines were grown on raised beds. While the breeding populations were unreplicated, the full-sibs populations were evaluated in two replications. A mixture of virulent races collected from leaf samples that were naturally infected in Agua Fria was used for inoculation as described in Singh et al. (2018a) and He et al. (2020). Four checks including Sonalika (susceptible check), Ciano T79 (susceptible check), Chirya.3 (resistant check) and Francolin #1 (moderately resistant check) were used. The spot blotch severity was scored using the double-digit scale (00–99) for rating foliar diseases, where the first digit represents the vertical progression of the disease and the second digit represents the severity of the disease (Saari and Prescott 1975; Eyal et al. 1987). Four to five evaluations in weekly intervals were done for each of the panels between the last week of January and the first week of March.

Evaluations of spot blotch in the KC and WC populations in Dinajpur and the CC population in Varanasi are described in Gahtyari et al. (2021) and Roy et al. (2021), respectively. For all the populations, the percentage disease severity and the relative area under the disease progress curve (rAUDPC) (Simko and Piepho 2012) values, where the AUDPC was expressed relative to the most susceptible line (whose rAUDPC was assumed to be 100) were obtained from the double-digit scores. In addition to spot blotch, we also recorded the days to heading (when about 50% of the plants in a plot had fully emerged spikes) and plant height (in cm) for all the panels in all the environments.

Outliers in the spot blotch rAUDPC values were detected using the Huber’s robust fit outliers method (Huber and Ronchetti 2009) and values that were more than four (*K* = 4) spreads from the center were considered as missing. Statistical analysis of the spot blotch rAUDPC values was done and the mean and standard deviation in the different panels were obtained. The Pearson’s correlation between the spot blotch rAUDPC values, days to heading and plant height, along with the *p-*values for the test of significance of the correlations were also obtained and visualized using the ‘R’ package ‘ggplot2’ (Wickham 2009) for the breeding panels. The spot blotch phenotyping data for the breeding panels is available in Supplementary Table 1 and the phenotyping data for the full-sibs panels has been reported before (Singh et al. 2018a; He et al. 2020; Gahtyari et al. 2021; Roy et al. 2021).

### Genotyping

Genome-wide genotyping-by-sequencing (GBS) markers (Poland and Rife 2012; Glaubitz et al. 2014) were obtained for all the seven breeding panels. We used the TASSEL v5 (Trait Analysis by aSSociation Evolution and Linkage) GBS v2 pipeline (Glaubitz et al. 2014) for calling the marker single nucleotide polymorphisms (SNPs) and Bowtie2 (Langmead and Salzberg 2012) to anchor 6,075,743 unique GBS tags to the first version of the reference sequence assembly of Chinese Spring (RefSeq v1.0) (IWGSC 2018). The SNPs were filtered using Fisher’s exact test, Chi-squared values and inbred coefficients as described in Juliana et al. (2019) and the resulting 78,606 SNPs were further filtered for missing data (less than 50%), minor allele frequency (greater than 5%) and heterozygosity (less than 5%). This resulted in 7918, 7503, 9695, 9873, 8130, 11648 and 9507 markers in breeding panels 2014–2020, respectively. We also filtered the lines in each breeding panel for less than 50% missing genotyping data and obtained 904, 949, 990, 1011, 962, 943 and 977 lines in panels 2014–2020, respectively.

All the four full-sibs panels were genotyped using the DArTseq GBS platform (Li et al. 2015) at the Genetic Analysis Service for Agriculture in Mexico, as described by Singh et al. (2018a)﻿. In addition, the lines were also genotyped for the *Vrn-A1* and the *Rht-B1* gene-based markers and these markers were added to the DArTseq markers dataset for analysis. Marker filtering for missing data, minor allele frequency and heterozygosity was done similar to the breeding panels, and we obtained 4278, 4079, 2707 and 3025 markers for the BC, CC, KC and WC full-sibs populations, respectively. Similarly, we also filtered lines with missing data greater than 50%, resulting in 231, 226, 224 and 231 lines in the BC, CC, KC and WC populations, respectively.

### Spot blotch prediction within panels

Prediction of spot blotch rAUDPC values within the breeding and full-sibs panels was done using a fivefold cross-validation approach, where each of the panels was divided into five random folds and four-fifth of the lines (training population) was used to predict the spot blotch breeding values of the remaining one-fifth of the lines (validation population). These random folds were sampled independently 20 times and the mean and standard deviation of the prediction accuracies (Pearson’s correlation between the spot blotch rAUDPC values and the estimated breeding values from different prediction models) across the 20 repetitions were obtained for all the datasets in the breeding and full-sibs panels.

Given the significant correlations between days to heading and spot blotch rAUDPC values in all the breeding panels, we generated subsets of the breeding panels after excluding early and late lines by treating them as outliers using the Huber’s robust fit outliers method (*K* was assumed to be 1) (Huber and Ronchetti 2009). This resulted in subsets with 657, 629, 469, 704, 676, 443 and 638 lines in the breeding panels 2014–2020, respectively (4216 lines in total). Similarly, significant correlations of days to heading and plant height with spot blotch rAUDPC values were observed in several full-sibs panel datasets. However, we did not generate full-sibs panel subsets, as removing the early and late lines in the full-sibs panels resulted in subsets of about 100 sibs, which is not considered as a reasonable population size to evaluate genomic prediction. Hence, it is to be noted that in the full-sibs panels used, spot blotch predictions also involved predicting the effects of the days to heading and plant height associated loci on the disease severity. For the lines within the breeding and full-sibs panels, we evaluated the following models:

1. Genomic-best linear unbiased prediction (GBLUP)
  Genomic prediction for spot blotch within the breeding and full-sibs panels was done using the GBLUP model that was fitted in the ‘R’ package ‘rrBLUP’ (Endelman 2011) and represented as:
  1 $${\varvec{y}} = 1\mu + {\varvec{Z}}_{g} {\varvec{u}} + {\varvec{\varepsilon}}{ }$$
  where *y* was the vector of spot blotch rAUDPC values, $\mu$ was the mean, ${\varvec{u}}$ was the additive genetic effects, *Z* was the design matrix for the random effects and ε was the error term. We assumed that the joint distribution of the vector of additive genetic effects (${\varvec{u}}$) and error term (***ε***) was multivariate normal i.e., $MN \left( {0, {\mathbf{G}}\sigma_{g}^{2} } \right)$ and $MN \left( {0, {\mathbf{I}}\sigma_{e}^{2} } \right)$. Here, **G** indicates the genomic relationship matrix calculated from markers (VanRaden 2008), σ^2^_g_ and σ^2^_e_ indicate the genetic and error variance, respectively and **I** indicates the identity matrix.
2. Fixed effects model
  The fixed effects model with selected markers significantly associated with spot blotch as fixed effects was fitted in the ‘R’ statistical software. First, the significant markers were identified using genome-wide association mapping in TASSEL version 5 (Bradbury et al. 2007) using the mixed linear model (Yu et al. 2006), where population structure accounted by the first two principal components was used as a fixed effect, and kinship was used as a random effect. Then, a *p*-value threshold of 0.0001 was used to declare significance of the markers to be included in the fixed effects prediction model, which resulted in 29, 10, 238, 15, 164, 12 and 28 significant markers in panels 2014 to 2020, respectively, that were reported in Juliana et al. (2022). However, since it is unrealistic to use over 100 markers in a fixed effects model, we used a stringent *p*-value threshold of 0.00001 for panel 2016 and panel 2018, that resulted in 64 and 84 significant markers, respectively. The spot blotch associated significant markers that were used as fixed effects in the different breeding panels are given in Supplementary Table 2. Similarly, for the subsets of breeding panels, we used a *p*-value threshold of 0.0001 to declare significant markers. This resulted in 25, 11, 38, 14, 41, 36 and 36 significant markers that were used as fixed effects in subsets of panels 2014–2020, respectively (Supplementary Table 3).
  For spot blotch predictions using the fixed effects model in the full-sibs panels, we used a stepwise least-squares approach (Heffner et al. 2011; Juliana et al. 2017a), where genome-wide association analysis was conducted in the training sets to identify markers that were significantly associated with spot blotch in different full-sibs panels. The marker p-values were obtained, and the markers were ranked based on their *p*-values, followed by which marker selection among the ranked markers was done using the following stepwise regression model:
  2 $${\varvec{y}} = 1_{n} \mu + {\varvec{X}}_{i} \beta_{i} \ldots {\varvec{X}}_{j} \beta_{j} + {\varvec{\varepsilon}}$$
  where *y* was the vector of spot blotch rAUDPC values, $\mu$ was the mean, ${X}_{i}$ and ${X}_{j}$ were the ith and jth marker’s genotype matrix and ${\beta }_{i}\mathrm{ and }{\beta }_{j}$ were the effects of the ith and jth marker. Then, for each iteration *i* through *j*, a marker was added to the model, with the first marker being the one that had the lowest *p*-value. The fivefold cross validation accuracies were then calculated within the training set after each iteration and the model with *j* − 1 markers was selected when the accuracy_j-1_ was greater than accuracy_j_. The selected markers (one to five markers in each fold given in Supplementary Table 4) were then used for the estimation of marker effects that were used for obtaining the estimated breeding values for spot blotch in the validation population.
3. Genomic-best linear unbiased prediction and fixed effects (GBLUP + fixed effects)
  In the GBLUP + fixed effects model, the markers that were used as fixed effects in the fixed effects models in the breeding and full-sibs panels were used in combination with markers fitted as random effects in the GBLUP model, and the model can be represented as:
  3 $${\varvec{y}} = 1_{n} \mu + {\varvec{X}}_{i} \beta_{i} \ldots {\varvec{X}}_{j} \beta_{j} + {\varvec{Z}}_{g} {\varvec{u}} + {\varvec{\varepsilon}}$$
  where all the terms were similar to those described in (1) and (2).
  We compared the prediction accuracies obtained from the different models and tested if they were significantly different from each other using paired-*t* tests. The mean differences in the prediction accuracies between the different model pairs and the *p-*values for the test of significance of the mean differences were obtained. A *p-*value threshold of 0.005 was used to declare significance of the mean differences for both one and two-tailed *t*-tests.

### Population structure and spot blotch prediction across breeding and half-sibs panels

Population structure for all the 6736 lines in the breeding panels and 912 lines in the full-sibs panels was obtained using the first two principal components (Patterson et al. 2006; Price et al. 2006) in TASSEL version 5. We then visualized the two principal components and clustering of different panels based on them, using the ‘R’ package ‘ggplot2’ (Wickham 2009). In the breeding panels, for predicting across panels, we used all other breeding panels to predict a given panel. Hence, the seven breeding panels with 904–1011 lines were predicted from large training sets of 5725–5832 lines comprising all the other panels. Similarly, in the subsets of breeding panels, 443–704 lines were predicted from training sets of 3512–3773 lines, comprising all the other subset panels.

Considering the four full-sibs panels, the full-sibs in each of them were related as half-sibs with the other panels, due to the common susceptible parent. Hence, we used all other half-sibs (681–688 half-sibs) to predict a given full-sibs panel with 224–231 full-sibs. For this, we used the full-sibs evaluations in Agua Fria in the 2012–2013, 2013–2014 and 2014–2015 cycles (where all the full-sibs panels were evaluated) and 9268 filtered DArTseq markers.

We also performed fivefold cross validations using random folds in the 6736 lines from all breeding panels, 4216 lines from all breeding panels subsets and 912 half-sibs, to understand if random sets of breeding lines and sibs can be predicted from the remaining individuals. For this, (1) in the breeding panels, 1347 random lines were predicted from a training population of the remaining 5389 lines, (2) in the breeding panels subsets, 843 random lines were predicted from a training population of the remaining 3373 lines and (3) in the sibs panels, 182 random full-sibs and half-sibs were predicted from the remaining 730 full-sibs and half-sibs. These random folds were sampled independently five times and the mean prediction accuracies were obtained.

Genomic prediction across the breeding and half-sibs panels was done using the GBLUP model (1). For predictions across breeding panels using the fixed effects (2) and the GBLUP + fixed effects models (3), we used 26 and 36 significant markers from Supplementary Tables 1 and 2, that were significant in more than one panel in the breeding panels and the subsets of breeding panels, respectively. Similarly, for predictions across half-sibs panels with the fixed effects and the GBLUP + fixed effects models, we used the marker selection approach and the stepwise regression model described before and one to eight markers were used as fixed effects for predicting each of the panels.

### Comparison of phenotypic and genomic selection for spot blotch

To compare PS with GS for spot blotch, we used two breeding panels (panel 2014 and panel 2018) where the correlations between the spot blotch rAUDPC values and days to heading were low, along with the subsets of these panels. We selected 10% of lines that had the lowest spot blotch rAUDPC values in these panels and also the lowest GEBVs obtained from the GBLUP model, for both within and across panel predictions. From the 10% lines that were selected by PS, we obtained the percentage of lines that were also selected by GS and the percentage of lines that were selected by PS only. From the remaining 90% of lines that were discarded by PS, we obtained the percentage of lines that were discarded by both PS and GS and the percentage of lines that were selected by GS only, but discarded by PS. Similarly for comparing PS with GS in the full-sibs panels, we used only the environments that had the highest within-panel genomic prediction accuracies for each full-sibs panel. This included the BC 2015 Agua Fria, CC 2016 Agua Fria, KC 2014 Agua Fria and WC 2015 Agua Fria datasets, where PS was compared to GS using both within and across-panel GEBVs.

### Selection indices for spot blotch, days to heading and plant height in half-sibs

To identify lines with low spot blotch severities and GEBVs that are also not late and tall, we used the 912 half-sibs from the four bi-parental populations that were all phenotyped for spot blotch in Agua Fria during three seasons (2012–2013, 2013–2014 and 2014–2015). We evaluated two selection index approaches: (1) the Eigen selection index method (ESIM) proposed by Cerón-Rojas et al. (2006) and (2) the linear phenotypic selection index (LPSI) proposed by Smith (1936), Hazel and Lush (1942) and Hazel (1943). While the ESIM approach considers the economic weights of the traits to be fixed but unknown, the LPSI considers the economic weights to be fixed but known.

In the ESIM index, the first Eigenvector is used as the criterion for the selection index with its elements determining the proportion of the trait that contributes to the selection index (Cerón-Rojas et al. 2006). The ESIM index can be represented as ***I ***$$=b'y$$***,*** where $${\mathbf{b^{\prime}}}$$$$= \left[ {b_{1} b_{2} \ldots b_{t} } \right]$$ is the unknown vector of coefficients and $${\textbf{y}^{\prime}} = \left[ {y_{1} y_{2} \ldots y_{t} } \right]$$ is a known vector of the trait’s phenotypic values, with ‘*t*’ being the number of traits (Cerón-Rojas et al. 2006; Céron-Rojas and Crossa 2018). The LPSI is a linear combination of several optimally weighted and observable trait values that permits the addition of extra merit in one trait in order to compensate minor defects in another trait (Hazel 1943; Céron-Rojas and Crossa 2018). The LPSI can be represented as $$I = \mathop \sum \nolimits_{i = 1}^{t} w_{i} h_{i}^{2} y_{i}$$, where $$w_{i}$$ is the ith economic weight and $$h_{i}^{2}$$ is the heritability of trait $$y_{i}$$ (Céron-Rojas and Crossa 2018).

The ESIM and the LPSI were used for selecting the top 10% of spot blotch resistant sibs that were not late and tall. We then obtained the expected genetic gain per trait for 10% selection using both the indices with the ‘RIndSel’ package in ‘R’ as described in Alvarado et al. (2018). We also obtained the selection response in terms of the selection differential which is the mean value of the phenotype for the individuals that were selected as parents expressed as a deviation from the mean value of the phenotype for the individuals in the parental generation before selection (Falconer and Mackay 1996). Because days to heading and plant height have no economic weights in breeding for spot blotch resistance, we assigned few arbitrary weights with different emphasis on these traits (i.e., − 0.2, − 0.4, − 0.6 and − 0.8) to understand the effect of these weights relative to a higher weight of − 1 for the spot blotch rAUDPC values and GEBVs in the LPSI index. The vectors of arbitrary weights (*w*′) for spot blotch rAUDPC values, GEBVs, days to heading and plant height evaluated using the LPSI index were *w*′ = [− 1 − 1 − 0.8 − 0.8], *w*′ = [− 1 − 1 − 0.6 − 0.6], *w*′ = [− 1 − 1 − 0.4 − 0.4] and *w*′ = [− 1 − 1 − 0.2 − 0.2].

## Results

### Phenotyping data

**Fig. 1:**
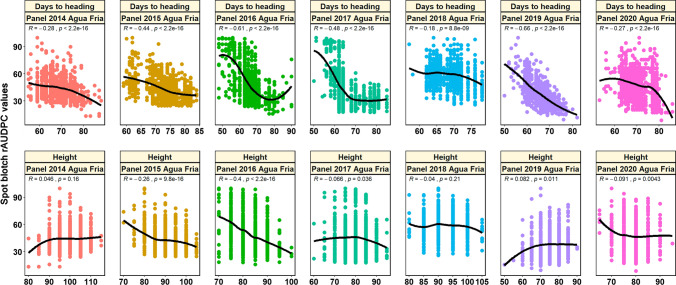
Scatter plot of the spot blotch relative area under the disease progress curve (rAUDPC) values, days to heading and plant height in panel 2014 (904 lines), panel 2015 (949 lines), panel 2016 (990 lines), panel 2017 (1011 lines), panel 2018 (962 lines), panel 2019 (943 lines) and panel 2020 (977 lines). The Pearson’s correlations between the traits and the *p*-values for the test of significance of the correlations are shown

In the breeding panels, the mean spot blotch rAUDPC values was the highest in panel 2018 (58.9 ± 12.8) and lowest in panel 2019 (37.5 ± 13.1). We observed significant (at a *p-*value threshold of 0.001) moderate to high negative correlations between the spot blotch rAUDPC values and days to heading, that ranged between − 0.18 and − 0.66 in different breeding panels (Fig. 1). However, considering the correlations of spot blotch rAUDPC values with plant height in the breeding panels, we observed that they were significant in only panel 2015 and panel 2016, while the strength and direction of the correlations were inconsistent ranging from moderately negative (− 0.40 in panel 2016) to weakly positive (0.08 in panel 2019) (Fig. 1).

In the full-sibs panels, the mean rAUDPC values ranged between 45.7 ± 15 (CC 2013 Varanasi) and 78.1 ± 12.8 (KC 2013 Dinajpur). The correlations between spot blotch rAUDPC values and days to heading were significant at a *p-*value threshold of 0.001 in all the full-sibs panels, except KC 2013 Dinajpur and CC 2013 Varanasi. Similarly, considering the correlations between the spot blotch rAUDPC values and plant height, we observed that they were significant at a *p-*value threshold of 0.001 in several panels.

### Spot blotch prediction accuracies within breeding panels

**Fig. 2:**
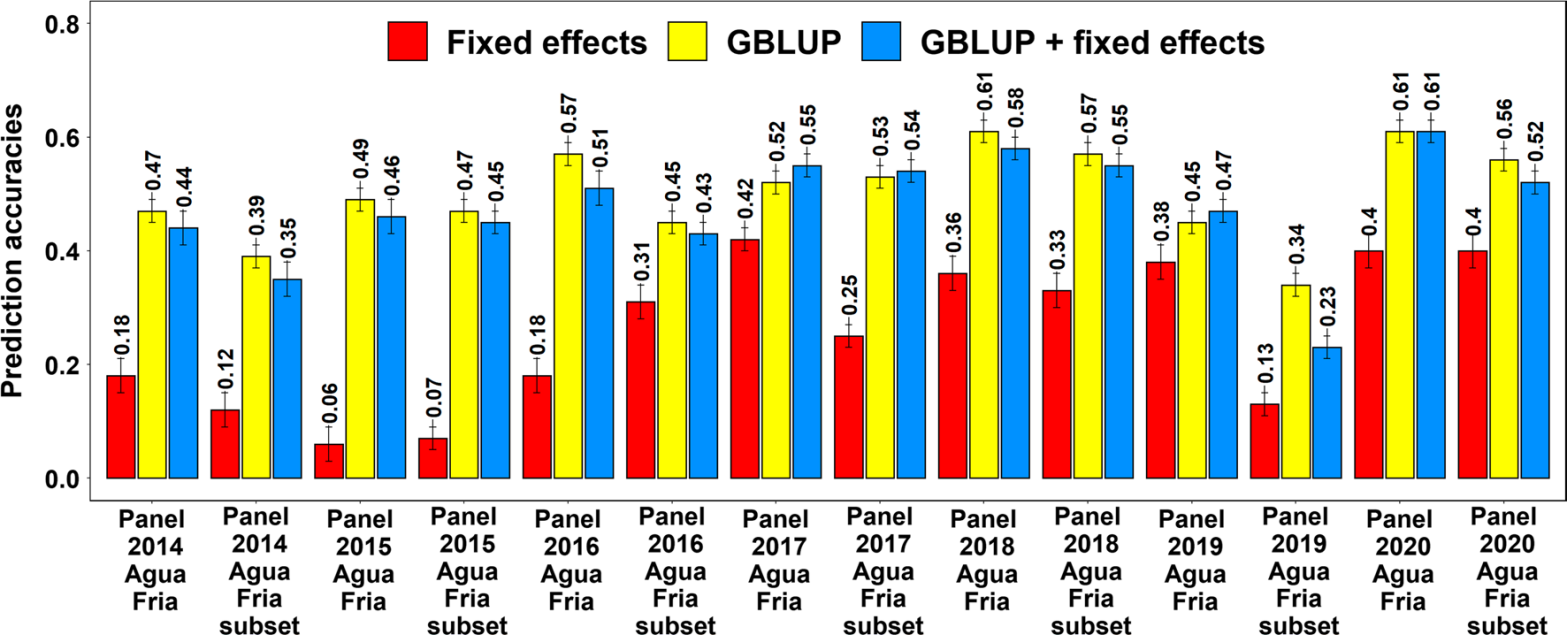
Mean spot blotch fivefold cross-validation prediction accuracies using the fixed effects model (with selected spot blotch associated markers as fixed effects), the genomic best linear unbiased prediction (GBLUP) model and the GBLUP + fixed effects model (GBLUP + fixed effects) in the breeding panels and subsets evaluated between 2014 and 2020 in Agua Fria. In the subsets of breeding panels, the early and late-heading lines were excluded

In the breeding panels with all the lines, the mean spot blotch prediction accuracy was the highest using the GBLUP model (0.53 ± 0.07), followed by the GBLUP + fixed effects model (0.52 ± 0.06) and the fixed effects model (0.28 ± 0.14) (Fig. 2). Similarly, in the subsets of breeding panels, the mean prediction accuracy was the highest using the GBLUP model (0.47 ± 0.09), followed by the GBLUP + fixed effects model (0.44 ± 0.12) and the fixed effects model (0.23 ± 0.12). Considering the breeding panels with all the lines and the subsets, we observed that: (1) the GBLUP model gave significantly higher prediction accuracies compared to the fixed effects model (mean difference = 0.25, *p-*value = 6.5E-07) (2) the GBLUP + fixed effects model gave significantly higher prediction accuracies compared to the fixed effects model (mean difference = 0.22, *p-*value = 9.8E − 07) and (3) the GBLUP and the GBLUP + fixed effects model accuracies were not significantly different (mean difference = 0.02, *p-*value = 2.2E − 02). We also observed that for within-panel predictions in both the breeding panels with all the lines and the subsets, the GBLUP gave a mean 177.6% increase in accuracy over the fixed effects model. To understand if prediction accuracies in the breeding panels with all the lines were different from those in the subsets of breeding panels, we tested the significance of the mean differences in prediction accuracies between them and observed that they were not significant using all the models.

### Spot blotch prediction accuracies within full-sibs panels

**Fig. 3:**
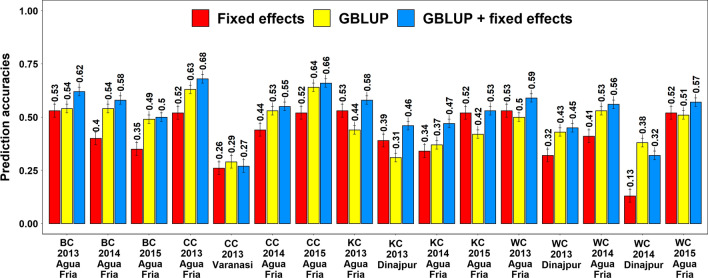
Mean spot blotch fivefold cross-validation prediction accuracies using the fixed effects model (with selected spot blotch associated markers as fixed effects), the genomic best linear unbiased prediction (GBLUP) model and the GBLUP + fixed effects model (GBLUP + fixed effects) in the full-sibs panels evaluated in Agua Fria, Varanasi or Dinajpur in growing cycles between 2013 and 2015. BC refers to the Bartai x Ciano T79 population, CC refers to the Cascabel x Ciano T79 population, KC refers to the Kath x Ciano T79 population and WC refers to the Wuya x Ciano T79 population

In within full-sibs panels predictions, the highest mean prediction accuracy was obtained using the GBLUP + fixed effects model (0.52 ± 0.11), followed by the GBLUP model (0.47 ± 0.10) and fixed effects model (0.42 ± 0.12) (Fig. 3). The mean differences in prediction accuracies within full-sibs panels were not significant for: (i) the GBLUP and the fixed effects models (mean difference = 0.05, *p-*value = 5E − 02) and (ii) the GBLUP + fixed effects and GBLUP models (mean difference = 0.05, *p-*value = 2.2E − 03). However, the mean difference in prediction accuracies (0.11) between the GBLUP + fixed effects and the fixed effects models was significant and the GBLUP + fixed effects model gave significantly higher accuracies compared to the fixed effects model in the full-sibs panels (*p-*value = 1.4E − 06). We also observed that for within full-sibs panels predictions, the GBLUP model gave a mean 22.1% increase in accuracy over the fixed effects model. In the different full-sibs panels, we observed that the genomic prediction accuracies using the GBLUP model were the highest in the BC panel (0.52 ± 0.03) and CC panel (0.52 ± 0.16), followed by the WC panel (0.47 ± 0.06) and KC panel (0.39 ± 0.06).

### Population structure and spot blotch prediction accuracies across the breeding panels

**Fig. 4:**
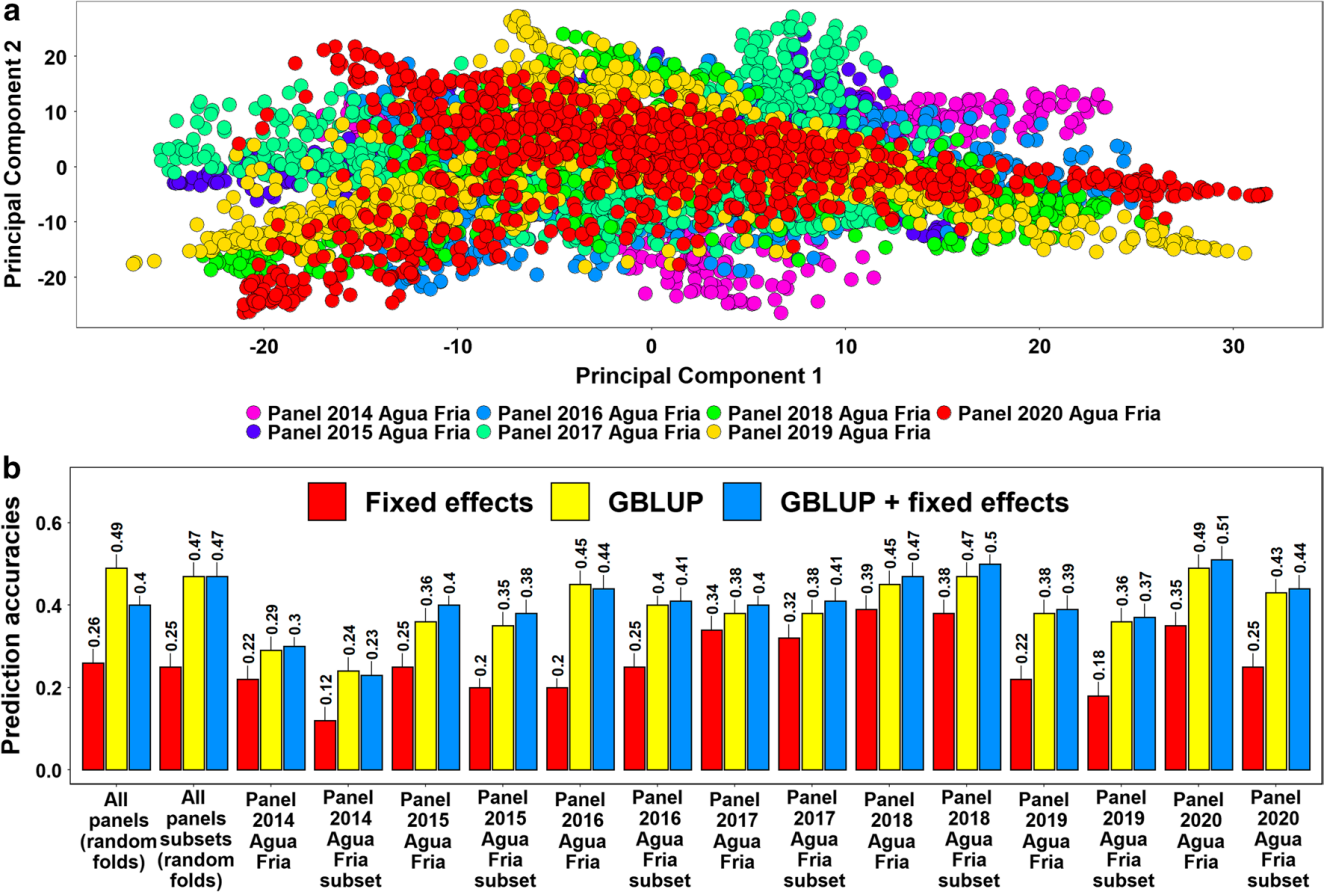
**a** Population structure in the breeding panels indicated by principal components 1 and 2. **b** Spot blotch prediction accuracies across the breeding panels and subsets evaluated between 2014 and 2020 in Agua Fria using the fixed effects model (with selected spot blotch associated markers as fixed effects), the genomic best linear unbiased prediction (GBLUP) model and the GBLUP + fixed effects model (GBLUP + fixed effects). The prediction accuracies for random folds indicate the fivefold cross validation accuracies using random folds in the breeding panel lines, where 1347 random lines were predicted from a training population of the remaining 5,389 lines and in the breeding panel subset lines, 843 lines were predicted from a training population of the remaining 3373 lines. In predictions across breeding panels, all the seven breeding panels with 904–1011 lines were predicted from large training sets of 5725–5832 lines comprising all the other panels. Similarly, in the subsets of breeding panels, 443–704 lines were predicted from training sets of 3512–3773 lines, comprising all other subsets

Population structure analysis indicated no clear clustering of the different breeding panels based on the first two principal components and substantial overlap between the lines from the different panels (Fig. 4a). Considering the prediction of individual breeding panels from all other breeding panels, we observed that the mean prediction accuracy was the highest using the GBLUP + fixed effects model (0.42 ± 0.07), followed by the GBLUP model (0.40 ± 0.07) and the fixed effects model (0.28 ± 0.08) (Fig. 4b). Similarly, in subsets of breeding panels, we observed that the GBLUP + fixed effects model (0.39 ± 0.08) resulted in the highest mean prediction accuracy across panels, followed by the GBLUP model (0.38 ± 0.07) and the fixed effects model (0.24 ± 0.09). In the random fivefold cross-validations across breeding panels, we observed that the highest accuracy in the complete set of breeding lines was obtained using the GBLUP (0.49) model, while in the subsets of lines, the highest accuracies were obtained using the GBLUP (0.47) and GBLUP + fixed effects (0.47) models.

In the breeding panels with all the lines and the subsets, we observed that the across-panel prediction accuracies from: (1) the fixed effects model was significantly lower than the GBLUP model (mean difference = 0.14, *p-*value for the one-sided *t*-test = 1.7E − 07) and the GBLUP + fixed effects (mean difference = 0.25, *p-*value for the one-tailed *t*-test = 6.5E − 07) model and (2) the GBLUP + fixed effects and the GBLUP models were not significantly different (mean difference = 0.01, *p-*value for the two-tailed *t*-test = 2.9E − 01). In across-panel predictions for the breeding panels with all the lines and the subsets, the GBLUP gave a mean 60.4% increase in accuracy over the fixed effects model. To understand if the across-panel spot blotch prediction accuracies of specific breeding panels were different from the prediction accuracies of random sets of lines across the breeding panels, we obtained the mean difference in prediction accuracies (0.04) between them for all the models and observed that they were not significant (*p-*value = 0.24).

### Spot blotch prediction accuracies across half-sibs panels

**Fig. 5:**
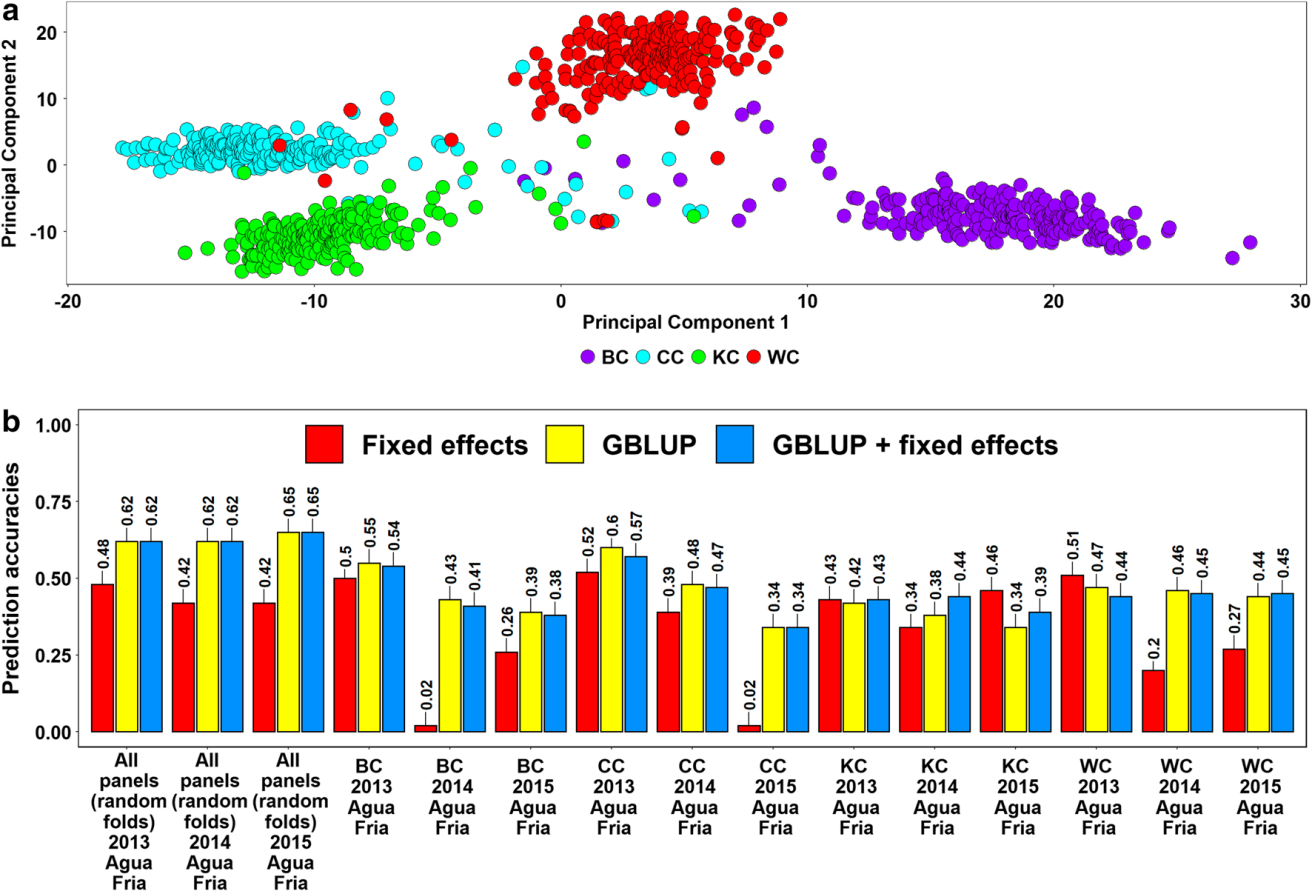
**a** Population structure in the sibs panels indicated by the principal components 1 and 2. BC refers to the Bartai x CianoT79 population, CC refers to the Cascabel × Ciano T79 population, KC refers to the Kath x  Ciano T79 population and WC refers to the Wuya x Ciano T79 population. **b** Prediction accuracies across the half-sibs panels evaluated between 2013 and 2015 in Agua Fria using the fixed effects model (with selected spot blotch associated markers as fixed effects), the genomic best linear unbiased prediction (GBLUP) model and the GBLUP + fixed effects model (GBLUP + fixed effects). The prediction accuracies for random folds indicate the fivefold cross validation accuracies when 182 random full-sibs and half-sibs were predicted from the remaining 730 full-sibs and half-sibs. In prediction across half-sibs panels, all other half-sibs panels comprising 681–688 lines were used to predict a given panel with 224–231 full-sibs

Population structure analysis of the sibs indicated clear clustering of different full-sibs panels based on the first two principal components and only a few sibs that could not be clearly differentiated (Fig. 5a). The mean prediction accuracy of a full-sib panel from all other half-sibs panels was the highest using the GBLUP + fixed effects model (0.44 ± 0.06) and the GBLUP model (0.44 ± 0.08), followed by the fixed effects model (0.33 ± 0.18) (Fig. 5b). In the prediction of random half-sibs and full-sibs using the remaining full-sibs and half-sibs, the highest mean accuracy was obtained using the GBLUP model (0.63 ± 0.02) and the GBLUP + fixed effects model (0.63 ± 0.02), followed by the fixed effects model (0.44 ± 0.03).

In predictions across half-sibs panels, we observed that the mean differences in prediction accuracies (ranged between 0 and 0.12) were not significant for all the model pairs (*p-*values ranged between 0.02 and 0.92). We also observed that for across half-sibs panels predictions (excluding BC 2014 Agua Fria and CC 2015 Agua Fria where the fixed effects model had accuracies close to 0), (1) the GBLUP gave a mean 26.7% increase in accuracy over the fixed effects model and (2) the GBLUP + fixed effects model gave a mean 0.9% increase in accuracy over the GBLUP model (ranged between 6.4% decrease and 15.8% increase). We also observed that using all the prediction models, the mean differences in prediction accuracies (ranged between 0 and 0.03) when the four full-sibs panels were predicted from other half-sibs panels were not significantly different (*p-*values ranged between 0.42 and 0.93).

### Comparison of phenotypic and genomic selection for spot blotch in the breeding panels

**Fig. 6:**
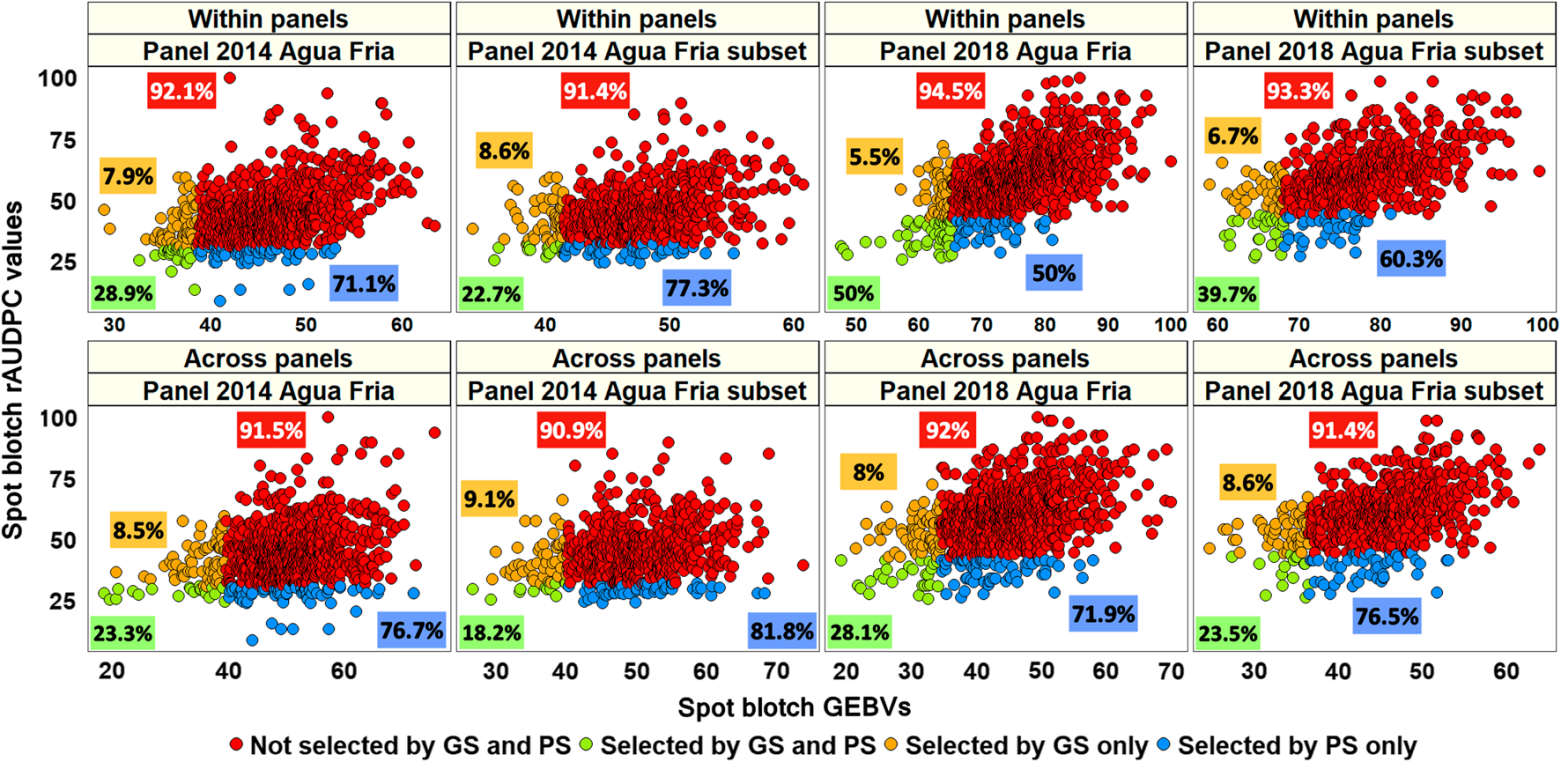
Comparison of phenotypic selection (PS) for spot blotch using the relative area under the disease progress curve (rAUDPC) values with genomic selection (GS) using the genomic estimated breeding values (GEBVs) obtained from the genomic best-linear unbiased prediction model in the breeding panels and subsets, for both within and across panel predictions. Among the lines that were discarded by PS, the percentages of lines that were not selected by GS and PS among the discarded lines are given in red boxes and the percentages of lines that were selected by GS only are given in orange boxes. Among the lines that were selected by PS, the percentages of lines that were selected by GS and PS are given in green boxes and the percentages of lines that were selected by PS only are given in blue boxes

In the 2014 and 2018 breeding panels with all the lines, when 10% of the resistant lines were selected, we observed that the mean percentage of lines that were not selected by GS and PS were 93.3 ± 1.7% in within-panel predictions and 91.8 ± 0.4% in across-panel predictions (Fig. 6). Similarly, the mean percentage of lines that were selected by both GS and PS were 39.5 ± 14.9% in within breeding panel predictions and 25.7 ± 3.4% in across breeding panel predictions. In the 2014 and 2018 breeding panels with subsets of lines, we observed that the mean percentage of lines that were not selected using both GS and PS were 92.4 ± 1.3% and 91.2 ± 0.4% in within and across panel predictions, respectively. Similarly, the mean percentage of lines that were selected using GS and PS in the breeding panels subsets were 31.2 ± 12% and 20.9 ± 3.7% in within and across panel predictions, respectively.

Furthermore, we also compared PS with GS, when the top 20%, 30%, 40% and 50% of the resistant lines were selected in the 2014 and 2018 breeding panels with all the lines. We observed that in within-panel predictions, the mean percentages of lines that were not selected by GS and PS were 86.4 ± 1.5%, 80 ± 3.03%, 74.1 ± 5.1% and 70 ± 5.3% when selecting the top 20%, 30%, 40% and 50% of the resistant lines, respectively. Similarly, in across-panel predictions, the mean percentages of lines that were not selected by GS and PS were 83.6 ± 1.2%, 76.2 ± 2.8%, 68.8 ± 4.7% and 60.5 ± 5.1%, when selecting the top 20%, 30%, 40% and 50% of the resistant lines, respectively. We also observed that in within-panel predictions, the mean percentages of lines that were selected by GS and PS were 45.5% ± 5.7%, 53 ± 7.2%, 61.2 ± 7.6% and 70 ± 5.3%, when the top 20%, 30%, 40% and 50% of the resistant lines were selected, respectively. In across-panel predictions, the mean percentages of lines that were selected by GS and PS were 34.2 ± 4.6%, 44.5 ± 6.6%, 53.3 ± 7% and 60.3 ± 5.2%, when the top 20%, 30%, 40% and 50% of the resistant lines were selected, respectively.

### Comparison of phenotypic and genomic selection for spot blotch in the full-sibs and half-sibs panels

**Fig. 7:**
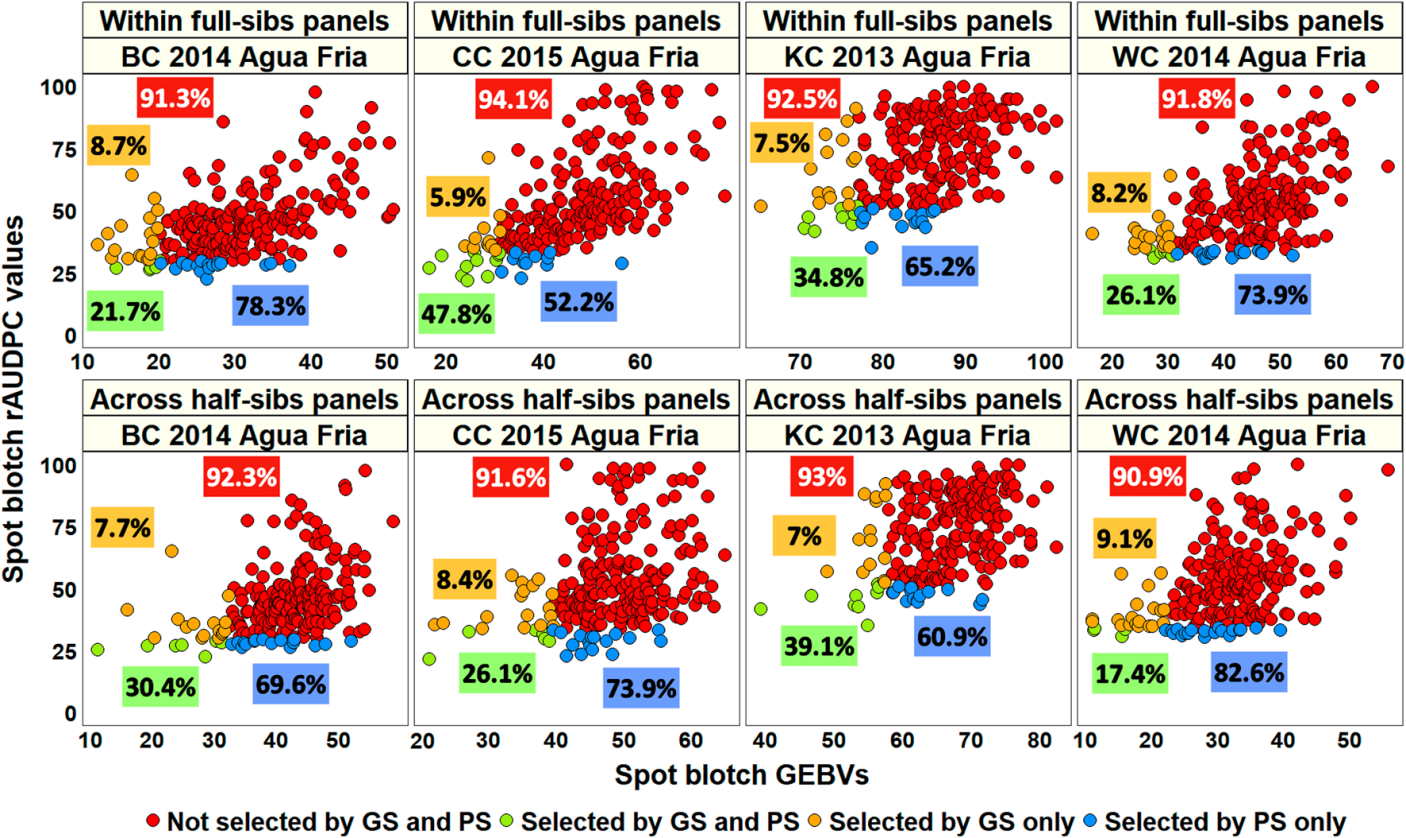
Comparison of phenotypic selection (PS) for spot blotch using the relative area under the disease progress curve (rAUDPC) values with genomic selection (GS) using the genomic estimated breeding values (GEBVs) obtained from the genomic best-linear unbiased prediction model in predictions within full-sibs panels and across-half sibs panels. BC refers to the Bartai x Ciano T79 population, CC refers to the Cascabel x Ciano T79 population, KC refers to the Kath x Ciano T79 population and WC refers to the Wuya x Ciano T79 population. Among the lines that were discarded by PS, the percentages of lines that were not selected by GS and PS among the discarded lines are given in red boxes and the percentages of lines that were selected by GS only are given in orange boxes. Among the lines that were selected by PS, the percentages of lines that were selected by GS and PS are given in green boxes and the percentages of lines that were selected by PS only are given in blue boxes

In predictions within full-sibs panels, we observed that the mean percentage of lines that were not selected by GS and PS were 92.4 ± 1.2% (Fig. 7). In predictions across half-sibs panels, we observed that the mean percentage of lines that were not selected by GS and PS were 92 ± 0.93%. The mean percentage of lines that were selected by both GS and PS were 32.6 ± 11.5% in within full-sibs panels predictions and 28.3 ± 9.1% in across half-sibs panels predictions.

### Selection indices for spot blotch, days to heading and plant height in half-sibs

The mean of all half-sibs, mean of selected 10% of half-sibs, selection differential and the expected genetic gain for 10% selected half-sibs are shown in Table 1. The selection differential for the spot blotch rAUDPC values ranged between − 18 (LPSI *w*′ = [− 1 − 1 − 0.8 − 0.8]) and − 19.2 (LPSI *w*′ = [− 1 − 1 − 0.2 − 0.2]), while the expected genetic gain for 10% selection ranged between − 18 (LPSI *w*′ = [− 1 − 1 − 0.8 − 0.8]) and − 21 (LPSI *w*′ = [− 1 − 1 − 0.2 − 0.2]). This indicated that a higher expected genetic gain for spot blotch rAUDPC values could be obtained with the lowest weights on days to heading and plant height (− 0.2). However, we also observed that an increase in emphasis on days to heading and plant height was inversely related to the expected genetic gain for spot blotch rAUDPC values.

**Table 1:** Mean of all half-sibs (912), mean of selected 10% of half-sibs (91), selection differential and the expected genetic gain for 10% selection for spot blotch relative area under the disease progress curve (rAUDPC) values, genomic-estimated breeding values (GEBVs), days to heading and plant height using the Eigen selection index method (ESIM) and the linear phenotypic selection index (LPSI) method

Estimated parameters	Spot blotch rAUDPC values	Spot blotch GEBVs	Days to heading	Plant height	Selection index
Mean of all half-sibs	56.7	63.6	62.1	99	
Mean of selected half-sibs	38.4	45.8	64.2	97.9	ESIM
	38.7	46.2	66	93.2	LPSI* w*′ = [− 1 − 1 − 0.8 − 0.8]
	38	45.8	66.6	95	LPSI * w*′ = [− 1 − 1 − 0.6 − 0.6]
	37.6	45.5	66.9	96.7	LPSI * w*′ = [− 1 − 1 − 0.4 − 0.4]
	37.5	45.2	67.3	99.2	LPSI * w*′ = [− 1 − 1 − 0.2 − 0.2]
Selection differential	− 18.3	− 17.8	2.1	− 1.1	ESIM
	− 18	− 17.4	3.9	− 5.8	LPSI * w*′ = [− 1 − 1 − 0.8 − 0.8]
	− 18.8	− 17.9	4.5	− 4	LPSI * w*′ = [− 1 − 1 − 0.6 − 0.6]
	− 19.1	− 18.1	4.8	− 2.3	LPSI * w*′ = [− 1 − 1 − 0.4 − 0.4]
	− 19.2	− 18.4	5.2	0.2	LPSI * w*′ = [− 1 − 1 − 0.2 − 0.2]
Expected genetic gain for 10%	− 19.4	− 15.4	3	2.7	ESIM
	− 18	− 15.4	3.8	− 2.7	LPSI * w*′ = [− 1 − 1 − 0.8 − 0.8]
	− 19.3	− 15.8	4.1	− 0.5	LPSI * w*′ = [− 1 − 1 − 0.6 − 0.6]
	− 20.3	− 15.9	4.3	1.6	LPSI * w*′ = [− 1 − 1 − 0.4 − 0.4]
	− 21	− 15.9	4.5	3.6	LPSI * w*′ = [− 1 − 1 − 0.2 − 0.2]

The different vectors of arbitrary weights (w') used in the LPSI index for spot blotch rAUDPC values, GEBVs, days to heading and plant height, respectively were *w′* = [− 1 − 1 − 0.8 − 0.8], *w*′ = [− 1 − 1 − 0.6 − 0.6], *w*′  = [− 1 − 1 − 0.4 − 0.4] and *w*′  = [− 1 − 1 − 0.2 − 0.2]

For the spot blotch GEBVs, we observed that the selection differential ranged between − 17.4 (LPSI *w*′ = [− 1 − 1 − 0.8 − 0.8]) and − 18.4 (LPSI *w*′ = [− 1 − 1 − 0.2 − 0.2]), while the expected genetic gain for 10% selected half-sibs ranged between − 15.4 (ESIM and LPSI *w*′ = [− 1 − 1 − 0.8 − 0.8]) and − 15.9 (LPSI *w*′ = [− 1 − 1 − 0.2 − 0.2] and LPSI *w*′ = [− 1 − 1 − 0.4 − 0.4]). For days to heading, the selection differential ranged between 2.1 (ESIM) and 5.2 (LPSI *w*′ = [− 1 − 1 − 0.2 − 0.2]), while the expected genetic gain for 10% selection ranged between 3 (ESIM) and 4.5 (LPSI *w*′ = [− 1 − 1 − 0.2 − 0.2]). We also observed that both the selection differential and the expected genetic gain for days to heading were positive, despite specifying negative weights. Considering plant height, we observed that the selection differential ranged between 0.2 (LPSI *w*′ = [− 1 − 1 − 0.2 − 0.2]) and − 5.8 (LPSI *w*′ = [− 1 − 1 − 0.8 − 0.8]), while the expected genetic gain for 10% selection ranged between 3.6 (LPSI *w*′ = [− 1 − 1 − 0.2 − 0.2]) and − 2.7 (LPSI *w*′ = [− 1 − 1 − 0.8 − 0.8]).

**Fig. 8:**
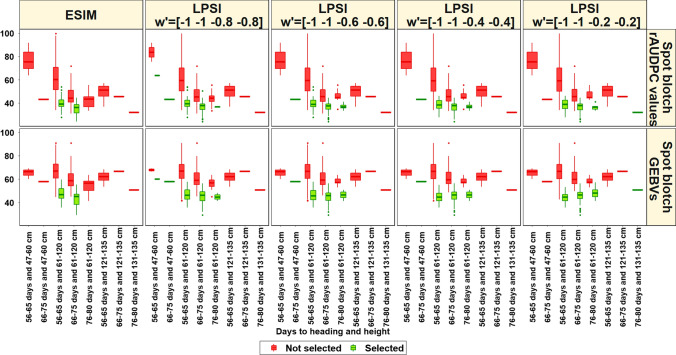
Box plots showing the distribution of spot blotch relative area under the disease progress curve (rAUDPC) values and genomic-estimated breeding values (GEBVs), days to heading and plant height for the 10% of the half-sibs that were selected using the Eigen selection index method (ESIM) and the linear phenotypic selection index (LPSI) and the remaining lines that were not selected. The different vectors of arbitrary weights (*w*′) used in the LPSI index for spot blotch rAUDPC values, GEBVs, days to heading and plant height, respectively were *w*′= [− 1 − 1 − 0.8 − 0.8], *w*' = [− 1 − 1 − 0.6 − 0.6], *w*′= [− 1 − 1 − 0.4 − 0.4] and *w*′= [− 1 − 1 − 0.2 − 0.2]

The box plots of trait distributions for the selected and unselected half-sibs using the ESIM and LPSI indices (Fig. 8) indicated that the ESIM only selected half-sibs that had low spot blotch rAUDPC values and GEBVs with normal days to heading and plant height (56–75 days and 61–120 cm). Using the LPSI *w*′ = [− 1 − 1 − 0.8 − 0.8] that laid a higher emphasis on days to heading and plant height some half-sibs with moderately high disease from the low days to heading and plant height (56–65 days to heading and 47–60 cm plant height) category were selected, which is undesirable. Similarly, with the LPSI *w*′ = [− 1 − 1 − 0.2 − 0.2] values that laid a low emphasis on days to heading and plant height, some half-sibs that had low disease, but were in the tall and late (76–80 days to heading and 131–135 cm plant height) category were also selected, which is also undesirable. The selections made by the LPSI *w*′ = [− 1 − 1 − 0.4 − 0.4] and LPSI *w*′ = [− 1 − 1 − 0.6 − 0.6] were quite similar to each other and to the ESIM, but the ESIM also resulted in the selection of lines with moderately high spot blotch, low days to heading and plant height (66–75 days and 47–60 cm) and late to normal plant height (76–80 days and 61–120 cm).

## Discussion

In this study, we first evaluated the scenario of using genomic prediction for spot blotch in bread wheat within advanced breeding panels, where we aimed to understand whether phenotyped random sets of four-fifths of the lines can be used to predict the spot blotch response in the remaining one-fifth of the lines in a panel/year. Our results indicated moderately high fivefold cross-validation mean prediction accuracies of 0.53 and 0.47 in the breeding panels and subsets of breeding panels, respectively, implying that genomic prediction for spot blotch within breeding panels is promising and can be implemented by breeding programs. In the second scenario, we evaluated genomic prediction across breeding panels to understand if a breeding panel can be predicted using historic training populations comprising other breeding panels. Our results indicated moderately high mean across-panel genomic prediction accuracies of 0.40 and 0.38 in the breeding panels and their subsets, respectively, which were only slightly lower than the corresponding within-panel cross-validation genomic prediction accuracies of 0.53 and 0.47. These results are in agreement with previous studies that have indicated inflated prediction accuracies using cross-validations within populations and the challenges in predicting across populations/years using historic training populations due to the lower proportion of shared marker effects or genomic relationships and a high genotype x environment interaction (Dawson et al. 2013; Jarquín et al. 2017; Juliana et al. 2018b, 2019; Werner et al. 2020; Haile et al. 2021). Nevertheless, the across-panel prediction accuracies for spot blotch were promising in our study. Hence when there is relatedness between the lines across breeding panels as demonstrated by our population structure analysis, historic training populations can be effectively used to predict the spot blotch response of a future unphenotyped panel of advanced breeding lines, albeit with moderate genomic prediction accuracies.

Genomic selection could be an effective selection tool for spot blotch resistance in generations prior to phenotyping where large-scale disease evaluation of sibs from multiple families is not possible due to the limited seed, excessive cost, logistical challenges and considerable resources involved. Hence, we have evaluated genomic prediction of sibs in full-sibs panels, half-sibs panels and a combined panel with full-sibs and half-sibs. Our results indicated that sibs were predicted with the highest mean accuracy (0.63) from a composite training population with random full-sibs and half-sibs. When full-sibs were predicted from other full-sibs within families and when full-sibs panels were predicted from other half-sibs panels, our results indicated mean prediction accuracies of 0.47 and 0.44, respectively. These are in agreement with previous studies suggesting that composite training sets with combined half-sibs families and a combination of half-sibs and full-sibs provide higher prediction accuracies compared to single families (Lehermeier et al. 2014; Brauner et al. 2020; Zhu et al. 2021). This is probably because of the higher number of sister lines in the training set and consistent linkage phases between the QTL associated with the trait and the markers in the half-sib families (Schulz-Streeck et al. 2012; Lehermeier et al. 2014).

Genomic prediction within full-sibs panels in a breeding program is challenging because the size of the training population in within-family predictions is limited. In addition, a separate training population has to be created for each cross, some full-sibs from each cross have to be phenotyped and not all full-sibs families are predicted with equally high prediction accuracies for a trait (Heffner et al. 2011; Juliana et al. 2020). Furthermore, prediction of a new family might not always result in high and positive prediction accuracies (Würschum et al. 2013; Lehermeier et al. 2014; Marulanda et al. 2015) and the best half-sib families that could predict another family are generally unknown in a breeding program (Zhu et al. 2021). Therefore, we suggest that using some full-sibs and half-sibs in the training population to predict other sibs is an ideal strategy for implementing GS for spot blotch resistance in early generations of the breeding program where there are a higher number of sibs compared to the advanced generations (Juliana et al. 2018a, 2020), and prediction of both the within-family Mendelian sampling term and the across-family parental average and Mendelian sampling term are involved (Daetwyler et al. 2007; Werner et al. 2020).

We tested the hypothesis that genomic prediction for spot blotch resistance would perform better than prediction using few selected loci as fixed effects in the breeding panels and full-sibs panels for both within and across-panels predictions. In the breeding panels with all the lines and the subsets, genomic prediction using the GBLUP model resulted in significantly higher accuracies, which were 177.6% and 60.4% higher than the mean accuracies from the fixed effects model, in within and across panel predictions, respectively. These results indicate that genomic prediction for spot blotch using genome-wide markers is superior to using few selected markers as fixed effects in the breeding panels, as hypothesized. They are also in agreement with previous studies that have reported higher accuracies with genomic prediction models compared to the fixed effects model (Meuwissen et al. 2001; Heffner et al. 2011; Rutkoski et al. 2012, 2014; Juliana et al. 2017b, a). The relatively lower prediction ability of the fixed effects model further affirms the quantitative and additive genetic inheritance of spot blotch resistance in bread wheat (Sharma et al. 1997; Kumar et al. 2010; Singh et al. 2018a; Gahtyari et al. 2021; Juliana et al. 2022).

In predictions within full-sibs panels and across half-sibs panels, we observed that the mean differences in prediction accuracies between the GBLUP and fixed effects models were not significant, but quite inconsistent, as the GBLUP gave accuracies that were 26.1% lower to 192.3% higher than the accuracies from the fixed effects model. The similarity in accuracies obtained using both the fixed effects model and genomic prediction is expected, given the large effects of the *Vrn-A1* gene and other markers on chromosome 5A that were used as fixed effects in several within full-sibs panels predictions and also reported to be linked with spot blotch response in linkage mapping studies in these full-sibs panels (Singh et al. 2018a; He et al. 2020; Gahtyari et al. 2021; Roy et al. 2021). These results also agree well with previous studies that have reported similar accuracies using the fixed effects model and genomic prediction, when the trait is controlled by large-effect loci (Juliana et al. 2017a; Emebiri et al. 2021). However, it should be noted that the prediction accuracies for spot blotch in the sibs reported in this study may be inflated, because they resulted from the large effects of the days to heading and plant height associated loci on spot blotch severity. Hence further studies in panels of sibs where days to heading and plant height have no association with spot blotch response are needed to understand the predictability of spot blotch *per se*.

Although the mean difference in accuracies from the GBLUP + fixed effects and the GBLUP model were not significantly different in both within and across panel predictions in the breeding and full-sibs panels, the accuracies from the GBLUP + fixed effects model were 32.4% lower to 48.4% higher than those from the GBLUP model. While several previous studies have reported an increase in prediction accuracies using some markers as fixed effects in genomic prediction models (Rutkoski et al. 2014; Juliana et al. 2017a; Odilbekov et al. 2019; Sehgal et al. 2020; Alemu et al. 2021), we attribute the similar accuracies obtained from the GBLUP + fixed effects and the GBLUP model in several breeding panels to the lack of large effect loci affecting the trait. Fitting major genes as fixed effects in genomic prediction models is expected to increase the prediction accuracy only for traits that have a high heritability, are controlled by few major genes and each major gene accounted for more than 10% of the genetic variance (Bernardo 2014), which was not the case in several spot blotch breeding datasets used in this study.

Since prediction accuracies are not the only criteria for implementing GS, we further analyzed how GS compares to PS for selecting spot blotch resistant lines and discarding susceptible lines. When the top 10% of the resistant lines were selected, a mean of 93.3%, 91.8%, 92.4% and 92% lines that were discarded by PS were also discarded by GS using the GEBVs from within breeding panels, across breeding panels, within full-sibs panels and across half-sibs panels predictions, respectively indicating that GS could be an ideal tool to discard susceptible lines. However, we observed that 60.6–74.3%, of the lines were selected only by PS and not by GS in different datasets and hence by relying solely on GS and using a stringent selection intensity, there is a risk of losing resistant lines. These results are comparable to those obtained by Juliana et al. (2018b), who also reported similar results from evaluating GS for grain yield in bread wheat.

Our results indicate that it is important to integrate GS with PS in selection decisions to increase the accuracy and circumvent the shortcomings of both selection methods i.e., PS based on single environment/year evaluations might not be very accurate due to the non uniform disease pressure in some years, variable disease incidence, disease escape etc., and GS using models trained with single environment/year evaluations might be challenging for the same reasons as PS, in addition to the inability of models to predict the effects of rare alleles with large effects that are not present in the training population. Moreover, our study has also demonstrated that a higher overlap between the lines selected by both GS and PS can be obtained when a higher proportion of lines are selected i.e., when 50% of the lines were selected, the mean percentage of lines that were selected by both GS and PS in across and within-panel predictions ranged between 60.3 and 70%, respectively. However, it is worth noting that breeding programs do not always have the choice to advance 50% of the lines to the next generation and a higher proportion of lines selected also decreases the selection intensity.

This study reports the first successful evaluation of the LPSI and ESIM to simultaneously select for spot blotch, days to heading and height. We have also demonstrated for the first time that it is possible to select simultaneously on the spot blotch rAUDPC values and GEBVs, which can facilitate breeding programs to accurately select individuals based on their net genetic merit. Our results indicated that the LPSI *w*′ = [− 1 − 1 − 0.8 − 0.8] selected some half-sibs with moderately high disease from the low days to heading and plant height category and the LPSI *w*′ = [− 1 − 1 − 0.2 − 0.2] selected some half-sibs in the tall and late category with low disease, because of the high and low weights on days to heading and plant height, respectively, and hence both of them are not appropriate. While selections using the ESIM were close to the breeder’s expectations, selections using the LPSI *w*′ = [− 1 − 1 − 0.4 − 0.4] and LPSI *w*′ = [− 1 − 1 − 0.6 − 0.6] were also acceptable.

Our results also indicated that the selection differential and expected genetic gain for days to heading were positive. This could be attributed to the elimination of several early lines that had high disease in the index-based selection, resulting in a higher mean for days to heading in the selected lines. While the best index that can be constructed will be far from perfect because of the effects of the environment and non-additive gene action (Hazel 1943), further optimization of the weights for breeding pipelines depending on the traits of priority (for ex. whether early or normal heading lines, high or moderately resistant lines are preferred for a particular pipeline) is important. In conclusion, we have demonstrated the prospects of integrating GS and index-based selection in spot blotch resistance breeding and they can be effectively utilized to increase the selection accuracy, response to selection and genetic gain for spot blotch.

## Supplementary Information

Below is the link to the electronic supplementary material.

Table S1: Spot blotch phenotyping data for the seven breeding panels with the relative area under the disease progress curve (rAUDPC) values. Table S2: Markers used as fixed effects in the five folds of the fixed effects model in the seven breeding panels for within breeding panels predictions. Table S3: Markers used as fixed effects in the fixed effects model in the seven breeding panels for across breeding panels predictions. Table S4: Markers used as fixed effects in the five folds of the fixed effects model in the full-sibs for within full-sibs panels predictions. BC refers to the Bartai x Ciano T79 population, CC refers to the Cascabel × Ciano T79 population, KC refers to the Kath × Ciano T79 population and WC refers to the Wuya x Ciano T79 population. (XLSX 198 KB)

## Data Availability

The phenotyping data of the breeding panels used in this study is available in Supplementary Table 1.
